# Real-time single-proton counting with transmissive perovskite nanocrystal scintillators

**DOI:** 10.1038/s41563-023-01782-z

**Published:** 2024-01-08

**Authors:** Zhaohong Mi, Hongyu Bian, Chengyuan Yang, Yanxin Dou, Andrew A. Bettiol, Xiaogang Liu

**Affiliations:** 1 Key Laboratory of Nuclear Physics and Ion-beam Application (MOE), Institute of Modern Physics, Fudan University, Shanghai, China; 2 Centre for Ion Beam Applications, Department of Physics, National University of Singapore, Singapore, Singapore; 3 Department of Chemistry, National University of Singapore, Singapore, Singapore; 4 Division of Science, Yale-NUS College, Singapore, Singapore; 5 SZU-NUS Collaborative Innovation Center for Optoelectronic Science & Technology, Shenzhen University, Shenzhen, China; 6 Institute of Materials Research and Engineering, Agency for Science, Technology and Research, Singapore, Singapore

**Keywords:** Quantum optics, Imaging techniques

## Abstract

High-sensitivity radiation detectors for energetic particles are essential for advanced applications in particle physics, astronomy and cancer therapy. Current particle detectors use bulk crystals, and thin-film organic scintillators have low light yields and limited radiation tolerance. Here we present transmissive thin scintillators made from CsPbBr_3_ nanocrystals, designed for real-time single-proton counting. These perovskite scintillators exhibit exceptional sensitivity, with a high light yield (~100,000 photons per MeV) when subjected to proton beams. This enhanced sensitivity is attributed to radiative emission from biexcitons generated through proton-induced upconversion and impact ionization. These scintillators can detect as few as seven protons per second, a sensitivity level far below the rates encountered in clinical settings. The combination of rapid response (~336 ps) and pronounced ionostability enables diverse applications, including single-proton tracing, patterned irradiation and super-resolution proton imaging. These advancements have the potential to improve proton dosimetry in proton therapy and radiography.

## Abstract (web summary)

Current organic proton detectors have poor detection sensitivities due to low light yields and limited radiation toleration. Here the authors report a perovskite nanocrystal-based transmissive thin scintillator that can detect seven protons per second, enabled by radiative emission from biexcitons.

## Main

Cancer radiotherapy with high-energy ion beams, particularly proton beams, has expanded rapidly in recent decades because it enables the delivery of the maximum possible dose to the target whilst sparing healthy tissue from radiation, in contrast to conventional X-ray and γ-ray therapy^1–3^. The rising demand for accurate dose control in proton therapy has led to extensive research into proton dosimeters such as ionization chambers, silicon-based detectors and plastic scintillators^4–6^. The dosimeters must be capable of accurately quantifying the number of protons during therapy in real time, ideally at the single-proton level. However, because ionization chambers and silicon detectors are too bulky, a proton beam cannot penetrate through this kind of detectors and be delivered to the patient in real time. Plastic scintillators, particularly scintillating fibres, are normally used in an invasive way to record the dose in real time, although they suffer from limited detection efficiencies and radiation hardness. The development of reliable and non-invasive detectors for real-time and accurate proton counting remains a challenge.

An alternative strategy is to fabricate high-performance thin-film detectors^7^, which are transmissive to protons and enable simultaneous proton counting during radiotherapy. Protons have been detected using thin-film diamond detectors and organic scintillators^8,9^. However, thin diamonds are both difficult to fabricate and expensive, and thin organic scintillators have low scintillation yields due to the low atomic electron density in organic materials (Methods). This implies that scintillators composed of high-atomic number (high-*Z*) and high-density materials could be suitable for proton detection.

Metal halide perovskite materials, which comprise heavy atoms and have recently emerged for the detection of ionizing radiation^10,11^, can be used to enhance the stopping power of protons. Single-crystal perovskites have been shown to be effective for detecting X-rays, γ-rays, β-rays, α-particles and neutrons through either the direct conversion of radiation quanta to an electrical signal or indirect conversion to visible photons via scintillation^12–20^. One early example used a lead zirconium titanate crystal as a bulk thermometer for the measurement of proton-beam currents^21^. However, single-crystal perovskites are not feasible for the fabrication of thin-film detectors. By contrast, perovskite nanocrystals, particularly all-inorganic caesium lead halide (CsPbBr_3_), can be easily fabricated into large-scale thin films^22–24^. Moreover, these nanocrystals combine the merits of efficient scintillation, fast response, tunable emission, high sensitivity and stability to radiation fluxes, and superior spatial resolution for radiographic imaging^22,24,25^. A notable property of lead halide perovskites is their tolerance to ion beams^26–28^. We thus hypothesize that thin-film scintillators made entirely of inorganic CsPbBr_3_ nanocrystals can achieve single-proton counting accuracy in transmission mode.

## Instrumentation and proton-induced luminescence

**Fig. 1:**
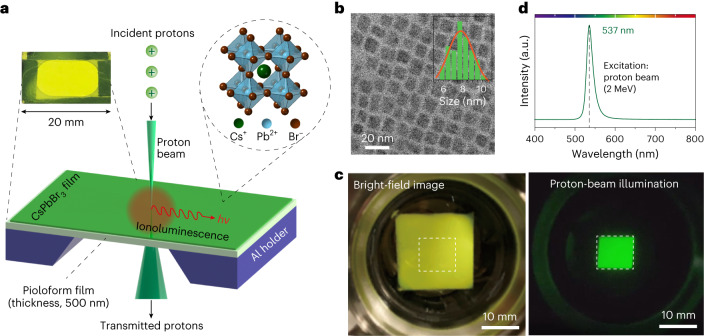
Proton-induced luminescence from CsPbBr_3_ nanocrystal scintillator. **a**, Schematic of proton-beam-induced luminescence (ionoluminescence) in a thin transmission scintillator comprising CsPbBr_3_ nanocrystals with a cubic crystal structure (shown by the upper right inset). The upper left inset shows an image of an actual CsPbBr_3_ scintillator prepared on a 500-nm-thick pioloform film. *hν*, photon energy; Al, aluminium. **b**, Representative transmission electron microscopy image of the as-synthesized CsPbBr_3_ nanocrystals. The inset histogram shows the size distribution of the nanocrystals. **c**, Experimental demonstration of proton-induced scintillation from a CsPbBr_3_ nanocrystal scintillator. The scintillator was pasted onto a glass viewport and placed inside a vacuum target chamber (10^−6^ mbar). Left: bright-field image of the scintillator. Right: proton-beam illumination within the square marked in the left image. **d**, Ionoluminescence spectrum of CsPbBr_3_ nanocrystals measured with proton-beam excitation at 2 MeV.

To validate our hypothesis, we used an ion accelerator with a hydrogen source to generate a proton beam with a typical kinetic energy of 2 MeV. Subsequently, the proton beam can be focused to a spot size of <30 nm through a nanoprobe-formation system that includes an objective aperture, a collimating aperture and a spaced triplet of magnetic quadrupole lenses^29^ (Supplementary Fig. 1). To allow incident protons to pass through, a thin scintillator made of CsPbBr_3_ nanocrystals was prepared on a 500-nm-thick pioloform film supported by a hollowed-out aluminium frame (Fig. 1a and Supplementary Fig. 2). The focused proton beam was scanned over the scintillator placed downstream along the beam trajectory and at the beam’s focal plane. The interaction of the protons with the scintillator produced ionoluminescence, a term used to describe luminescent photon emission upon ion-beam illumination^30^. The ionoluminescent light was collected with a customized aluminium parabolic mirror to maximize the light collection efficiency (Supplementary Fig. 1). The collected ionoluminescent light was then focused onto a photomultiplier tube (PMT) for single-photon counting.

The photoluminescence quantum yield (PLQY) of thin-film scintillators comprising surface-treated CsPbBr_3_ nanocrystals was measured to be approximately 71% (see Methods for details). Transmission electron microscopy imaging revealed that the as-synthesized CsPbBr_3_ nanocrystals had a typical cubic shape with an average lateral size of 7.8 nm (Fig. 1b and Supplementary Fig. 3). As such a size approaches the Bohr radius of nanocrystals, proton-induced excitons can be confined tightly within a nanocrystal through quantum confinement. Consequently, exciton recombination results in the emission of ionoluminescence photons. To demonstrate proton-induced scintillation from these CsPbBr_3_ nanocrystals experimentally, a scintillator was placed in a vacuum target chamber of protons (Fig. 1c). The nanocrystals scintillated upon proton illumination (using a beam current of ~100 pA at an energy of 2 MeV), and the ionoluminescence was imaged through a transparent glass viewport. Subsequently, the ionoluminescence was collected to measure the spectrum, which revealed an emission centred at 537 nm with an energy of 2.32 eV (Fig. 1d).

## Proton scintillation mechanism investigations

**Fig. 2:**
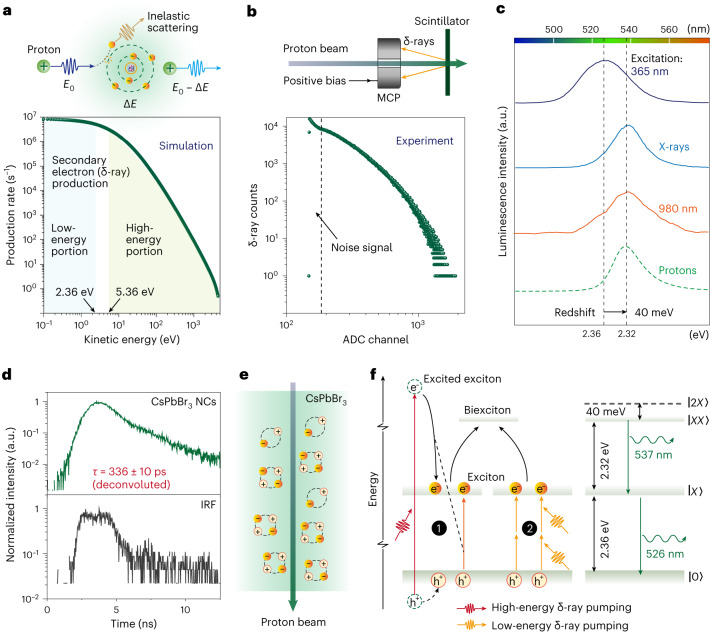
Mechanistic investigation of proton-induced luminescence in CsPbBr_3_ nanocrystals. **a**, Top: illustration of proton-induced atomic ionization in the production of secondary electrons, through energy deposition (Δ*E*) of an incident proton (of energy *E*_0_). Bottom: calculated energy distribution of secondary electrons (δ-rays) in a CsPbBr_3_ nanocrystal. The energy values of 2.36 eV and 5.36 eV correspond to the bandgap energy (*E*_g_) of the CsPbBr_3_ nanocrystal and the minimal energy required to form an electron–hole pair, respectively. **b**, Top: schematic of the experimental set-up for the measurement of back-emitted δ-rays produced by a proton beam (2 MeV) in a perovskite scintillator with a microchannel plate (MCP) detector. Bottom: the measured number of δ-rays in terms of their corresponding energy expressed via the channel number of the analogue-to-digital converter (ADC). **c**, Luminescence emission spectra of CsPbBr_3_ nanocrystals under excitation with a 365 nm light source, 40 keV X-rays (99 μA), a 980 nm laser and 2 MeV protons, respectively. **d**, Time-resolved ionoluminescence measurements. Note that the instrumental response function (IRF) was acquired by measuring the ionoluminescence response of a fast-decay material (Methods), and the decay time *τ* (~336 ps) was determined as a result of deconvolution with the IRF. NCs, nanocrystals. **e**, Schematic of biexcitons as four-body quasiparticles. Biexcitons are formed from excitons that are produced densely around the track of the proton beam. **f**, Proposed mechanism of proton scintillation in a CsPbBr_3_ nanocrystal. Process 1 represents multiple exciton generation via impact ionization. Process 2 represents the second route for multiple exciton generation via below-bandgap low-energy δ-ray pumping. The biexcitons are converted from the excitons formed via processes 1 and 2. Subsequently, de-excitation from the biexcitonic state (|*XX*〉) to the excitonic state (|*X*〉) results in the emission of ionoluminescence photons at 537 nm (2.32 eV). De-excitation from the excitonic state (|*X*〉) to the ground state (|0〉) results in 526 nm emission (*E*_g_, 2.36 eV). Note that the virtual state |2*X*〉 corresponds to an energy level of 2*E*_g_. e^−^, electron; h^+^, hole.

We next investigated the mechanistic processes that govern the scintillating ionoluminescence upon the interaction of energetic protons with CsPbBr_3_ nanocrystals. It has been established that protons interact mainly with atomic electrons in the target during matter penetration via inelastic scattering^31^. As a result, a penetrating proton loses its energy, a portion of which is transferred to ionized electrons to generate secondary electrons (δ-rays). We performed a theoretical calculation of the production frequency as a function of the kinetic energy of these δ-rays in CsPbBr_3_ nanocrystals using the Hansen–Kocbach–Stolterfoht model^31^. The calculation result indicated that the kinetic energy of these δ-rays varies from the subelectronvolt level to about 4.3 keV, whilst the δ-ray production frequency reduces rapidly with increasing energy (Fig. 2a). The theoretical prediction of δ-ray production was experimentally verified by measuring the back-emitted electrons from the surface of a CsPbBr_3_ scintillator sample (Fig. 2b). The energy distribution of the detected δ-rays has the same trend as the high-energy portion of the calculated result in Fig. 2a. This is reasonable considering the detection limit of the electron detector and that high-energy δ-rays are more likely to reach the detector. Moreover, we designed an additional experiment to confirm the production of charge carriers (electrons and holes) in the perovskite scintillator upon proton irradiation, by measuring the current flowing through two electrode pads deposited on the surface of the scintillator film (Supplementary Fig. 4).

To shed light on the excitation mechanisms of CsPbBr_3_ nanocrystals by these proton-induced δ-rays, we first measured the emission spectra of the same CsPbBr_3_ nanocrystal scintillator (with a thickness of 35.1 μm) under different types of excitation source (Fig. 2c). When excited using a 365 nm light source, the scintillator emitted at 526 nm with a full-width at half-maximum (FWHM) of 29 nm, corresponding to an energy of 2.36 eV that is assumed to be the bandgap energy (*E*_g_) of the CsPbBr_3_ nanocrystal. In this case, the emission is due to the recombination of excitons at the bottom of the conduction band, which occurs upon the above-bandgap absorption of 365 nm photons and subsequent rapid thermalization. Intriguingly, compared with the emission under 365 nm excitation, the emission induced by 2 MeV protons was redshifted (~537 nm) and had a narrower FWHM (~21 nm), which was the same as that under excitation with X-ray and near-infrared (NIR; ~980 nm) light (Fig. 2c).

To explore the origins of the redshifts observed in luminescence emission, we next measured the emission lifetimes of the CsPbBr_3_ nanocrystal scintillator under different excitation sources. The results indicated that the timescale of the decay under ultraviolet (UV) and NIR light excitation was the same, of the order of 10 ns (Supplementary Fig. 5). This is similar to the reported lifetime of CsPbBr_3_ nanocrystals under X-ray excitation^22^. In these cases, we attributed the redshifts to re-absorption, which occurs due to the deeper penetration depth of NIR light and X-rays than UV light. This was further supported by the absence of redshifts in the perovskite solution containing CsPbBr_3_ nanocrystals (Supplementary Fig. 6). Intriguingly, the emission lifetime of the CsPbBr_3_ nanocrystal scintillator under proton-beam excitation showed a distinct difference (~336 ps; Fig. 2d). This fast decay is a typical signature of multiexcitons in nanocrystals, resulting from a quite efficient Auger process^32–35^. Considering that re-absorption does not alter the emission lifetime notably, we speculate that the redshift (~40 meV) observed under proton-beam excitation results from the formation of multiexcitons, specifically biexcitons, which are four-body quasiparticles produced by the interaction of two excitons (Fig. 2e), given the larger magnitude of the redshift compared with trions^36^. A biexciton has a lower energy than two free excitons and features a sharply defined state^37,38^, which could explain the redshifted emission and narrower FWHM under proton excitation.

The above-bandgap absorption of optical photons leads to a gradual prolifieration of electron–hole pairs, whilst excitons formed within a short time along the track of megaelectronvolt protons can be more densely populated^39^. In particular, when the energy of the proton-induced δ-rays exceeds the minimal energy required to form an electron–hole pair (that is, (4.58*E*_g_ − 5.45) eV ≈ 5.36 eV)^10^ (Fig. 2a), the generation of multiple excitons through impact ionization could be dominant^40,41^. In such a regime, the high-energy portion of the δ-rays (depicted in Fig. 2a) pumps the CsPbBr_3_ nanocrystal to form a high-energy exciton. Instead of thermalization by phonon emission, the excited exciton relaxes to the band edge via energy transfer (≥1*E*_g_) to a valence-band electron to promote it to the conduction band. As a result, high-energy δ-ray pumping leads to two excitons. The pumping mechanism, known as impact ionization (process 1 of Fig. 2f), generates a dense population of the excitonic state (|*X*〉), facilitating the rapid conversion of two excitons to the biexcitonic state (|*XX*〉) within a short time domain^37,38^ (Fig. 2f).

Moreover, excitation of the CsPbBr_3_ nanocrystals by 980 nm photons, with an energy of about 0.5*E*_g_, involves a nonlinear below-bandgap absorption process^42,43^. Our further study indicated that this absorption was attributed to a two-photon upconversion process (Supplementary Fig. 7). In our previous work, we found that ion-beam-induced low-energy δ-rays can stimulate the upconversion processes in lanthanide-doped nanocrystals^30^. Given this observation and the fact that low-energy photons (980 nm) can drive the upconversion process in CsPbBr_3_ nanocrystals, we propose an upconversion mechanism involving the absorption of two or multiple δ-ray quanta that have appropriate below-bandgap energies (depicted in the low-energy portion of Fig. 2a). This mechanism provides another means of populating the excitonic state in CsPbBr_3_, as shown in process 2 of Fig. 2f.

As a result of pumping with both low-energy and high-energy δ-rays, the biexcitonic state of the CsPbBr_3_ nanocrystals can be populated. Subsequent de-excitation of the biexcitons to excitons gave rise to the redshifted emission at 537 nm (Fig. 2f). Notably, the presence of shallow trap states below the conduction band could also contribute to the redshifts in emission. However, the decay associated with shallow traps is considerably longer (above 100 ns). To rule out the possibility of shallow trap formation due to defects produced by megaelectronvolt proton irradiation, we conducted experiments, and the results confirm the absence of such traps (Supplementary Figs. 8 and 9).

## Performance characterization and single-proton counting

The fast response of CsPbBr_3_ nanoscintillators to proton beams (~336 ps) enables the avoidance of overlapping counting in situations where a vast number of protons are encountered in a short time. For instance, in clinical proton radiography, the typical proton flux is of the order of 10^6^ per second^44^, which corresponds to approximately 0.0003 protons within the decay time of the perovskite nanoscintillator. Therefore, a CsPbBr_3_ nanocrystal scintillator exhibits sufficient speed for the accurate counting of single protons.

**Fig. 3:**
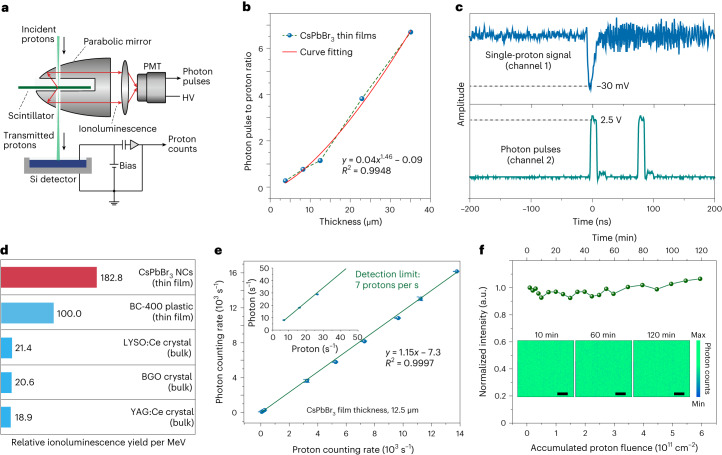
Performance characterization and single-proton counting with CsPbBr_3_ nanocrystals. **a**, Schematic of the experimental set-up for single-proton counting and calibration. A customized parabolic mirror, with two openings to allow the passage of the proton beam, was used to collect the ionoluminescence photons induced by protons from the CsPbBr_3_ scintillator. The collected light was focused onto a PMT for photon counting. A silicon (Si) detector was placed downstream of the proton beam to count the transmitted protons. HV, high voltage. **b**, Quantum yield of CsPbBr_3_ nanocrystal scintillators, characterized by the ratio of photon pulses to proton counts, as a function of the scintillator thickness. *R*^2^, coefficient of determination. **c**, Single-proton tracing in perovskite nanocrystal thin films (thickness, ~15 µm) using a fast oscilloscope (Tektronix MDO3024). The count rate of the incident protons was kept low (~500 s^−1^) to avoid overlap of the single-proton signals. The signal of a single proton (amplitude, −30 mV) and the corresponding signal of the proton-induced photon pulses (amplitude, 2.5 V) were recorded, respectively, from channels 1 and 2 of the oscilloscope. In this case, a single proton corresponds to a detectable yield of two photon pulses. **d**, Relative ionoluminescence light yield per MeV of a CsPbBr_3_ nanocrystal thin-film scintillator compared with a BC-400 plastic thin-film scintillator and (Lu,Y)_2_SiO_5_:Ce (LYSO:Ce), Bi_4_Ge_3_O_12_ (BGO) and Y_3_Al_5_O_12_:Ce (YAG:Ce) bulk crystal scintillators, under excitation by a 2 MeV proton beam (Methods). **e**, Measurement of the ionoluminescence photon counting rate as a function of the proton counting rate with a 12.5-µm-thick CsPbBr_3_ scintillator. The inset shows the ionoluminescence profiles measured at low proton counting rates, indicating a detection limit of 7 protons per s. *n* = 3 independent experiments. Data are presented as the mean ± the standard error of the mean. **f**, Ionoluminescence intensity profile as a function of the accumulated proton fluence, showing the pronounced ionostability of the perovskite scintillator (thickness, 50 μm). The inset shows ionoluminescence images taken at different time intervals (10, 60 and 120 min), which indicate that the emission brightness of the nanodiamonds remains essentially undiminished. Scale bars, 20 μm.

To assess the single-proton counting capability of ultrathin perovskite nanoscintillators, experiments were designed to simultaneously count proton-induced ionoluminescence photons and the transmitted protons (Fig. 3a). We prepared a set of thin-film CsPbBr_3_ scintillators with different thicknesses, via blade-coating, on 500-nm-thick pioloform membranes, and systematically investigated their responses to protons. Importantly, for each transmission scintillator with a certain thickness, the detectable photon pulse was linearly proportional to the proton count within the measurement range of ~10^2^ to 10^5^ protons (Supplementary Fig. 10). The ratio of ionoluminescence photon pulses to protons increased with the thickness of the scintillator (Fig. 3b). The curve fitting result indicated that the light output is approximately proportional to Δ*E*^3/2^, where Δ*E* represents the energy loss of protons, which is proportional to the scintillator thickness (Supplementary Fig. 11). This result agrees well with the empirical formula developed for organic scintillators^45^. To validate the single-proton counting capability of the CsPbBr_3_ nanocrystal thin films, we performed a direct demonstration by tracing the signals of a single proton and the corresponding photon pulses induced by this proton using a fast oscilloscope (Fig. 3c and Supplementary Fig. 12). In this case, only one proton was present within a time domain of 400 ns, and the proton counting rate was maintained at a sufficiently low level (~500 s^−1^).

To evaluate the scintillation sensitivity of the perovskite nanocrystals to proton beams, we compared the relative ionoluminescence yield per megaelectronvolt of the CsPbBr_3_ nanocrystal thin films (thickness, ~5 µm) with that of several widely used commercially available proton scintillators (Fig. 3d). The perovskite thin-film scintillators exhibited an ionoluminescence photon conversion efficiency for proton-beam energy that is approximately double that of commercially available BC-400 plastic thin-film scintillator (with a thickness of ~15 µm). This efficiency is approximately ten times greater than that typically observed in bulk scintillators such as LYSO:Ce, BGO and YAG:Ce crystals. Considering that the YAG:Ce crystal scintillator has a light yield of 3 × 10^4^ photons per MeV under proton beams^46^, we can estimate the light yield of the CsPbBr_3_ nanocrystal thin-film scintillator to be 2.9 × 10^5^ photons per MeV under proton beams. This noteworthy proton scintillation sensitivity of the CsPbBr_3_ nanocrystals is highly advantageous for detecting the lowest detectable proton flux, which was found to be 7 protons per s (Fig. 3e). Notably, this value is about five orders of magnitude lower than the proton flux used for clinical proton radiography^44^.

Moreover, the CsPbBr_3_ scintillator exhibited superior photon emission stability under proton irradiation (Fig. 3f). The ionoluminescence remained undiminished over 2 h with an accumulated fluence of up to 6 × 10^11^ protons per cm^2^, which is about seven orders of magnitude higher than a typical fluence used in proton radiography^44^, and two orders of magnitude higher than that used for proton therapy^2^. It is worth noting that the ionoluminescence intensity remained stable until the proton fluence reached 5 × 10^14^ cm^−2^ (Supplementary Fig. 13).

## Single-proton irradiation and high-resolution imaging

**Fig. 4:**
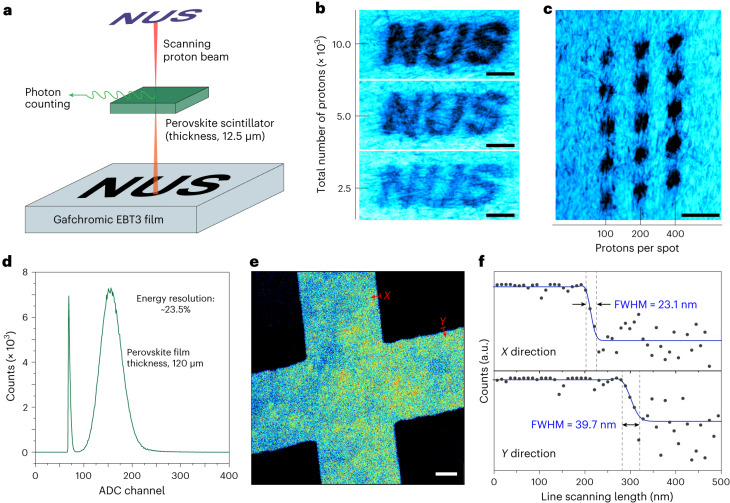
Single-proton irradiation and high-resolution proton imaging using the CsPbBr_3_ nanocrystal scintillator. **a**, Schematic of simultaneous single-proton counting and irradiation using a Gafchromic EBT3 film. The scanning of the proton beam (2 MeV) was patterned in the form of the letters ‘NUS’, where the irradiated region of the Gafchromic EBT3 film turned dark. The darkness is related to the number of irradiated protons. In this case, the thickness of the perovskite scintillator film was 12.5 µm. **b**, Optical imaging of the Gafchromic EBT3 film irradiated with the ‘NUS’ pattern. The images from bottom to top correspond to irradiation with 2,500, 5,000 and 10,000 protons, respectively. Scale bars, 30 μm. **c**, Optical imaging of the Gafchromic EBT3 film irradiated with a spot array. The columns of irradiated spots from left to right correspond to irradiation with 100, 200 and 400 protons per spot, respectively. Scale bar, 30 μm. **d**, Energy-resolved spectrum of 2.1 MeV protons detected with a 120-μm-thick CsPbBr_3_ scintillator coupled to a PMT. The signal was processed using an amplifier with a shaping time of 0.5 µs. Note that the ADC channel number is proportional to the energy of the protons. **e**, False-coloured image showing two crossed bars of a nickel grid (thickness, 2 μm), taken by measuring the energy loss of protons as they penetrate the grid pixel by pixel using the CsPbBr_3_ scintillator. Scale bar, 1 μm. **f**, Cross-sectional line profiles extracted along the arrows in the *X* and *Y* directions shown in **e**, indicating a lateral resolution of 23.1 nm horizontally and 39.7 nm vertically for proton imaging with the scintillator. The FWHM values were obtained by fitting the profiles using a modified Gaussian function (Methods).

CsPbBr_3_ nanocrystals, owing to their fast response, ultrahigh sensitivity and pronounced stability under proton irradiation, together with the proton transmission capability of the ultrathin perovskite scintillators, enable controllable single-proton irradiation in real time. As a proof of concept, a Gafchromic EBT3 radiation-sensitive film was utilized to receive the dose of accumulated single protons. In this case, the radiation-induced effects were visible and the simultaneous counting of single protons was realized using the CsPbBr_3_ scintillator (thickness, ~12.5 µm), which was transmissive to the proton beam (Fig. 4a). The scanning of the focused proton beam was patterned to deliver a certain number of single protons into the Gafchromic EBT3 film along a pre-set irradiation shape. As a demonstration, the series of letters ‘NUS’, with colours from light to dark corresponding to an increasing number of implanted protons, was clearly visualized through optical imaging (Fig. 4b). Moreover, a spot-scan pattern of the proton beam on the Gafchromic EBT3 film indicated directly the ability of the perovskite scintillator to count single protons (Fig. 4c). Notably, the proton beam dwelled at each irradiation spot until a certain number of protons had been counted using the scintillator.

We took a step further and assessed the suitability of CsPbBr_3_ nanocrystals as scintillators for high-resolution proton imaging. In this case, a thicker scintillator film (~120 μm) was prepared and used to ensure that the energy of the incident protons is fully deposited in the scintillator (Methods and Supplementary Fig. 14). Figure 4d shows a representative energy-resolved spectrum when the scintillator was exposed to a beam of 2.1 MeV protons. An energy resolution of 23.5% was measured from the FWHM of the spectrum. With such an energy-resolving power, the scintillator was used further to discriminate the energy loss of single protons as they passed through a home-made nickel calibration grid^29^ (Supplementary Fig. 15). An image of the internal structure of the grid was created by measuring the energy loss of transmitted protons pixel by pixel (Fig. 4e). The lateral resolution values of the image were 23.1 nm horizontally and 39.7 nm vertically, suggesting that CsPbBr_3_ nanocrystals are excellent candidates for high-resolution proton imaging (Fig. 4f).

In conclusion, we have demonstrated CsPbBr_3_ nanocrystals as a new class of scintillators that are highly suitable for high-sensitivity detection and counting of single accelerated protons. Our experimental and theoretical findings suggest that proton scintillation arises primarily from population of the biexcitonic state of the nanocrystal, through the pumping of proton-induced δ-rays. As a result, the CsPbBr_3_ nanocrystals exhibit fast and stable ionoluminescence emission with much higher light yields than commercially available proton scintillators. These physical characteristics make it possible to develop ultrathin and flexible perovskite scintillators with CsPbBr_3_ nanocrystals for real-time ultrasensitive single-proton counting. Importantly, these properties offer the possibility of accurate dose control at the single-proton level when used in proton-irradiation applications. Although several challenges need to be addressed before clinical use as proton dosimeters, these CsPbBr_3_ nanocrystal scintillators offer overwhelming promise for advancing the emerging detection technology in proton therapy and radiography.

## Methods

### Synthesis of CsPbBr_3_ nanocrystals

CsPbBr_3_ perovskite nanocrystals were synthesized using a modified hot-injection procedure^22,47^. Afterwards, an aqueous solution of ammonium fluorosilicate (AHFS; 200 μl, 0.5 M), obtained by dissolving 5 mmol AHFS in ultrapure water (10 ml), was added to a toluene solution of the CsPbBr_3_ nanocrystals (15 ml, 5 mg ml^−1^) to form AHFS–CsPbBr_3_ nanocrystals^48^. Details of the synthesis of the CsPbBr_3_ nanocrystals can be found in the Supplementary Methods. The PLQY of the pristine CsPbBr_3_ nanocrystals was measured to be approximately 63%, and the PLQY of the AHFS-treated CsPbBr_3_ nanocrystals was measured to be ~81%. These PLQY measurements were conducted using an integrating sphere (Edinburgh Instruments) with the excitation monochromator set at 365 nm.

### Fabrication of thin-film CsPbBr_3_ nanocrystal scintillators

In our study, thin-film CsPbBr_3_ nanocrystal scintillators were synthesized at room temperature. The sediment of the AHFS–CsPbBr_3_ nanocrystals (0.35 g) was first dissolved in toluene (5 ml). The solution of AHFS–CsPbBr_3_ nanocrystals was then concentrated to 3 ml under vacuum. Subsequently, the solution of AHFS–CsPbBr_3_ nanocrystals (500 ml) was taken and coated onto a 500-nm-thick pioloform film supported by a custom hollowed-out aluminium holder using a Zehntner ZAA2300 film coater. A thin-film scintillator comprising CsPbBr_3_ nanocrystals was finally formed via the natural volatilization of the toluene solvent at room temperature. A series of thin-film perovskite scintillators with various thicknesses was fabricated following these procedures by controlling amount of AHFS–CsPbBr_3_ nanocrystal solution used.

### Instrumentation for focused proton-beam formation

The megaelectronvolt proton beams used in this study were produced using a single-ended 3.5 MV electrostatic ion accelerator (HVE Singletron). A radio-frequency ion source was inbuilt in the accelerator along with a hydrogen bottle. To demagnify the proton beam, an optical system successively included a set of object apertures controlled using differential micrometers (OM10, Oxford Microbeams) to determine the initial lateral size of the beam, a series of collimation apertures placed downstream to control beam aberrations, a spaced triplet of magnetic quadrupole lenses (OM52, Oxford Microbeams) to focus the beam and a vacuum target chamber to host the beam focal plane^29^. The proton beam was scanned electrostatically using a module with parallel *X* and *Y* plates driven by two high-voltage amplifiers (Trek 609E-6 or AE Techron 7224). The input signal of the amplifiers was controlled using an IonDAQ system hosted by a computer^49^.

### Physical characterization

Ionoluminescence spectra were measured using a high-sensitivity spectrometer (QE65000, Ocean Optics) excited using 2 MeV protons. Ultraviolet-visible absorption spectra were measured using an Agilent Cary Series spectrophotometer (Shimadzu UV-2450). Photoluminescence and radioluminescence spectra were obtained using a fluorescence spectrometer (FLS1000, Edinburgh Instruments) equipped with a Xe900 lamp, a nanosecond-pulsed optical parametric oscillator laser (Ekspla) and a miniature X-ray source (Amptek). Time-resolved photoluminescence under UV light excitation was measured using time-correlated single-photon counting and a multi-channel scaling board (TimeHarp 260-PICO-Dual, PicoQuant) coupled to a 405 nm picosecond pulsed laser and a Nikon inverted microscopy. Time-resolved photoluminescence under NIR light excitation was collected using a pair of lenses in a spectrometer (Acton Spectra Pro 2500i) and detected using a charge-coupled device (Pixis 400B, Princeton Instruments), which was measured using an Optronis Optoscope Streak camera system. The 980 nm pump laser was generated using an 800 nm OPerA Solo coherent optical parametric amplifier. The 800 nm fundamental pulse with a pulse width of around 150 fs originated from a 1 kHz regenerative amplifier and was seeded by a mode-locked titanium:sapphire oscillator. All optical measurements were performed at room temperature. Transmission electron microscopy imaging and energy-dispersive X-ray spectroscopy were carried out using a JSM-2010F JEOL transmission electron microscope. Crystal structures of the CsPbBr_3_ nanocrystals were examined using an X-ray powder diffractometer (Bruker D8 ADVANCE Eco) using Cu Kα radiation (wavelength, *λ* = 1.5406 Å).

### Proton-induced δ-ray measurements

To measure the back-emitted δ-rays upon proton irradiation (2 MeV), an annular MCP (Hamamatsu) was used to collect the electrons that escaped from the surface of the perovskite scintillator by applying a bias voltage of 350 V to the front plate and an acceleration voltage of 2,800 V at the back plate. The MCP was equipped with a home-made pre-amplifier and a shaping amplifier (Ortec 672) for signal processing. The processed signal was distributed to the different channels of an ADC based on the pulse height of the signal, which is proportional to the energy of the collected electrons. To confirm the production of δ-rays within the perovskite scintillator upon proton irradiation, an electron–hole extraction device was designed by coating a thin layer of CsPbBr_3_ nanocrystals (50 μm thick) onto an Al_2_O_3_ ceramic substrate and sputtering two coplanar 50-nm-thick gold electrodes (100 μm apart) onto the scintillator film using a magnetron sputtering machine. Current–voltage characterization of the device was performed using a Keithley 2400 source meter.

### Thickness measurement of the perovskite films

The interaction of megaelectronvolt protons with a thin perovskite film results in the loss of proton energy *E*, predominantly through an electronic stopping process in which a penetrating proton interacts mainly with the atomic electrons of the target sample. The electronic energy loss (d*E*) of a proton when penetrating a distance of d*x* can be described by the Bethe–Bloch formula as follows:

$${\left(-\frac{{{\mathrm{d}}E}}{{{\mathrm{d}}x}}\right)}_{{\mathrm{e}}}=\sum _{i}\left\{\frac{4\uppi {Z_{1}}^{2}{e}^{4}}{{m}_{{\mathrm{e}}}{v}^{2}}{N}_{i}{Z}_{2i}\left[\ln\left(\frac{2{m}_{{\mathrm{e}}}{v}^{2}}{{I}_{i}}\right)\right]+\ln\left(\frac{1}{1-{\beta }^{2}}\right)-{\beta }^{2}-\frac{C}{{Z}_{2i}}\right\}$$

where *v* and *Z*_1_ represent the speed and atomic number of the impinging proton, respectively; *N*_*i*_, *Z*_2*i*_ and *I*_*i*_ are the number density, atomic number and mean ionization potential of the *i*th target atom, respectively; *m*_e_ represents the rest mass of the electron; *e* represents the elementary charge; *C* is a variable describing the shell correction; and *β* = *v*/*c*, where *c* is the speed of light in a vacuum. The Bethe–Bloch formula implies that high-*Z* and high-density scintillators are capable of gaining energy from protons, resulting in high scintillation yields.

For a thin film with a thickness *t* and mass density *ρ*(*x*) at a depth of *x* along the proton path, the relationship between *t* and the stopping power is as follows:

$${\int }_{0}^{t}\rho (x)\,{{\mathrm{d}}x}={\int }_{{E}_{0}}^{{E}_{{\mathrm{f}}}}{\left[\frac{{{\mathrm{d}}E}}{{\mathrm{d}}\left(\,\rho (x)x\right)}\right]}^{-1}\,{{\mathrm{d}}E}$$

where *E*_0_ and *E*_f_ represent the initial energy and the remaining energy of the proton, respectively, after penetrating the sample. In our case, the mass density of the perovskite scintillator film is 4.56 g cm^−3^. We assumed a constant mass density (*ρ* = 4.56 g cm^−3^), and the film thickness was obtained by experimentally measuring the energy loss of protons and calculating the stopping power using the SRIM software package^50^.

### Time-resolved ionoluminescence measurements

If the count rate of protons is low enough, one can assume that a proton beam is discretized into single protons. For example, in a time domain of 10 ns—the typical decay time of CsPbBr_3_ nanocrystals under optical excitation—less than one proton can be detected at a count rate of 10,000 protons per s. Using a proton-transmissive perovskite nanocrystal thin film as a scintillator, a single proton penetrated the thin scintillator and was detected using a silicon surface barrier detector (Ortec). The signal from this single proton was amplified to about 30 mV using a fast pre-amplifier (Diamond TCT Amplifier, Cividec Instrumentation; 2 GHz, 40 dB). The amplified single-proton signal was then captured using a fast oscilloscope (Tektronix MDO3024). The transistor–transistor logic signal (5 V) was then triggered by the oscilloscope. This transistor–transistor logic signal was used as the start signal for synchronization with the time-correlated single-photon counting hardware (TimeHarp 260 PICO, PicoQuant). A hybrid photomultiplier detector (PMA Hybrid 40, PicoQuant) coupled with a bandpass filter was used to detect single ionoluminescent photons emitted from the CsPbBr_3_ nanocrystals. A single-photon statistical histogram was formed over multiple cycles by registering the photon arrivals per time bin (50 ps). A schematic of the time-resolved ionoluminescence measurement system is provided in Supplementary Fig. 16.

In contrast to the pulsed-laser-based lifetime measurement system, it is not feasible to obtain the IRF of the time-resolved ionoluminescence measurement system directly. In our approach, we first used poly[2-methoxy-5-(2-ethylhexyloxy)-1,4-phenylenevinylene] (MEH-PPV) films, from which fast decay (~140 ps) was reported^51^, to measure the ionoluminescence decay profile. This decay profile was considered as the convolution of an exponential decay function *f*_1_(*x*) with the Gaussian IRF *f*_2_(*x*), as follows:

$$f(x)={y}_{0}+({\,f}_{1}\times {f}_{2})(x)={y}_{0}+\frac{A}{{t}_{0}}{e}^{\frac{1}{2}{\left(\frac{w}{{t}_{0}}\right)}^{2}-\frac{x-{x}_{{\mathrm{c}}}}{{t}_{0}}}{\int }_{-\infty }^{z}\frac{1}{\sqrt{2\uppi }}{e}^{-\frac{{y}^{2}}{2}}\,{\mathrm{d}}y$$

where ${f}_{1}\left(x\right)=\frac{A}{{t}_{0}}{e}^{-\frac{x}{{t}_{0}}}$, ${f}_{2}\left(x\right)=\frac{1}{w\sqrt{2\uppi }}{e}^{\frac{{\left(x-{x}_{{\mathrm{c}}}\right)}^{2}}{2{w}^{2}}}$ and $z=\frac{x-{x}_{{\mathrm{c}}}}{w}-\frac{w}{{t}_{0}}$, *y* is a variable dependent on *x*, and *y*_0_, *A*, *t*_0_, *w* and *x*_c_ are fitting parameters. In this way, the IRF can be obtained via calculation with the known decay time of the MEH-PPV films. Next, we measured the ionoluminescence decay profile of the CsPbBr_3_ nanocrystals. Subsequently, through a deconvolution procedure, the decay time of the CsPbBr_3_ nanocrystals could be determined. Data processing for both convolution and deconvolution was performed using OriginPro software (version 2021b, OriginLab Corporation).

### Measurement of the photon-to-proton ratio

Single ionoluminescence photons induced by the proton beam in a perovskite scintillator were collected using a customized parabolic mirror and detected with a PMT (Hamamatsu R7400P) equipped with a photon counting unit (Hamamatsu C9744). Single protons transmitted through the thin scintillator were detected using a silicon surface barrier detector (Ortec). The numbers of ionoluminescence photons and transmitted protons were recorded using the IonDAQ acquisition system via two ADC channels to determine the photon-to-proton ratio.

### Measurement of the relative ionoluminescence yield

The ionoluminescence yield of the scintillators used in our study depends on many factors, such as the light collection efficiency, the PMT detection efficiency, the energy deposition and the beam-current fluctuation of the incident protons. Therefore, it is difficult to determine the absolute ionoluminescence light yields of the scintillators. Instead, we were able to perform relative ionoluminescence yield measurements. Using the same light collection and detection geometry^52^, we acquired the number of ionoluminescence counts using the PMT (Hamamatsu R7400P) and the proton counts for each scintillator using the silicon surface barrier detector (Ortec). For thin-film scintillators (CsPbBr_3_ nanocrystals and BC-400 plastic), the energy deposition of protons in the films was determined using the method described in Supplementary Fig. 11. For the bulk scintillators (YAG:Ce, BGO and LYSO:Ce), the energy deposition was equal to the energy of the proton beam (2 MeV). The number of ionoluminescence counts was calibrated using the spectral quantum efficiency of the PMT for different emission wavelengths. Subsequently, these counts were normalized by the respective number of protons and the energy deposition specific to each scintillator. The respective normalized number of ionoluminescence counts per megaelectronvolt for the perovskite thin-film scintillator, the YAG:Ce crystal scintillator, the BGO crystal scintillator and the LYSO:Ce crystal scintillator was then compared with that of the BC-400 plastic scintillator, whose normalized ionoluminescence yield was set to 100 per MeV.

### Single-proton irradiation and imaging with perovskite scintillators

The ratio of ionoluminescence photons and transmitted protons in a 12.5-μm-thick perovskite scintillator was first calibrated and determined before single-proton irradiation of the Gafchromic EBT3 films. The counting of single impinging protons was performed by measuring the ionoluminescence photon number induced by the thin scintillator. The scan patterns for proton irradiation were realized by deflecting the proton beam via control software (Ionscan 6.0)^53^. The irradiated Gafchromic EBT3 films were imaged using a Nikon optical microscope equipped with a ×20 objective. To measure the energy resolution of the perovskite scintillator (120 μm thick) when detecting 2.1 MeV protons, the PMT (Hamamatsu R7400P) was connected to the shaping amplifier (Ortec 672) with a shaping time of 0.5 µs and a gain of 200. The processed signal was then recorded by the IonDAQ acquisition system via an ADC to obtain the energy-resolved spectrum.

For proton imaging, the scintillator (120 μm thick) was placed behind a calibration grid (thickness, 2 µm; bar width, 4 µm; see Supplementary Fig. 8). The energy difference of the transmitted protons passing through the different areas of the grid can be discriminated by the scintillator due to the different numbers of ionoluminescence photons. Therefore, a pulse height of the PMT, which is proportional to the number of detected photons, is inversely proportional to the energy loss of the protons when penetrating the grid. The pulse-height signal was then recorded from a 1024 × 1024 pixel array to map the density of the grid. Notably, to eliminate the imaging noise caused by beam-current fluctuation, 15 pulse-height counts were recorded at each pixel and the median pulse-height signal was selected as the pulse height of that pixel. The edge profiles of the grid image were then fitted to obtain the lateral resolution of proton imaging using a modified Gaussian function^54^:

$$y=A\left[1+{{\mathrm{erf}}}\left(2\sqrt{\ln2}\frac{a-x}{f}\right)\right]+B\exp \left[-\ln16{\left(\frac{a-x}{f}\right)}^{2}\right]+C$$

where *A*, *B* and *C* are unitless fitting parameters, erf is the Gaussian error function, *a* is a fitting parameter indicating the position of the bar edge and *f* represents the FWHM.

## Online content

Any methods, additional references, Nature Portfolio reporting summaries, source data, extended data, supplementary information, acknowledgements, peer review information; details of author contributions and competing interests; and statements of data and code availability are available at 10.1038/s41563-023-01782-z.

### Supplementary information

Supplementary Information Supplementary Methods and Figs. 1–16.

## Data Availability

The datasets that support the findings of this study are available from the corresponding authors upon reasonable request.
