# Supplementary material for: Real-time single-proton counting with transmissive perovskite nanocrystal scintillators
Source: Nat Mater. 2024 Jan 8;23(6):803–9. doi: 10.1038/s41563-023-01782-z (PMC11150155; doi:10.1038/s41563-023-01782-z)
Supplement: Supplementary file 1 — Supplementary Methods and Figs. 1–16. [file 41563_2023_1782_MOESM1_ESM.pdf]

nature materials

Article https://doi.org/10.1038/s41563-023-01782-z

Real-time single-proton counting with transmissive perovskite nanocrystal scintillators

In the format provided by the authors and unedited

Table of Contents

Supplementary Methods……………………………………………………………………………S2

Supplementary Fig. 1……………………………………………………………………………S4

Supplementary Fig. 2……………………………………………………………………………S4

Supplementary Fig. 3……………………………………………………………………………S5

Supplementary Fig. 4……………………………………………………………………………S6

Supplementary Fig. 5……………………………………………………………………………S7

Supplementary Fig. 6……………………………………………………………………………S7

Supplementary Fig. 7……………………………………………………………………………S8

Supplementary Fig. 8……………………………………………………………………………S9

Supplementary Fig. 9……………………………………………………………………………S10

Supplementary Fig. 10………………………………………………………………………...S11

Supplementary Fig. 11………………………………………………………………………...S12

Supplementary Fig. 12………………………………………………………………………...S13

Supplementary Fig. 13………………………………………………………………………...S14

Supplementary Fig. 14………………………………………………………………………...S15

Supplementary Fig. 15………………………………………………………………………...S16

Supplementary Fig. 16………………………………………………………………………...S17

Supplementary Methods

Materials. Cesium carbonate (Cs_2CO_3, 99.9%), lead(II) bromide ($PbBr_2$, 99.99%), cadmium oxide (CdO, 99.99%), zinc acetate ($Zn(CH_3CO_2)_2$, 99.99%), selenium (Se, 99.99%), sulfur (S, 99.98%), trioctylphosphine ($[CH_3(CH_2)_7]_3P$, 97%), oleic acid (technical grade 90%), 1-octadecene (technical grade 90%), oleylamine (technical grade 70%), ammonium hexafluorosilicate (AHFS, $(NH_4)_2SiF_6$, 98%), and poly[2-methoxy-5-(2-ethylhexyloxy)-1,4-phenylenevinylene] (MEH-PPV) were purchased from Sigma-Aldrich. All chemicals were used without further purification.

Synthesis of Cs-oleate as a Cesium Precursor. In a typical synthesis procedure, Cs_2CO_3 (1.628 g, 5 mmol), oleic acid (10 mL) and octadecene (10 mL) were added to a two-neck round-bottomed flask (100 mL). The resulting mixture was heated to 100 °C for 0.5 h with vigorous stirring under vacuum, and stirred for another 0.5 h under vacuum environment at 100 °C. Subsequently, the mixture was heated to 120 °C in a nitrogen atmosphere and the solution became clear. The clear solution indicated that the reaction between Cs_2CO_3 and oleic acid was complete. The Cs-oleate precursor solution was kept at 80 °C in a nitrogen atmosphere before the synthesis of $CsPbBr_3$ nanocrystals.

Synthesis of $CsPbBr_3$ nanocrystals. In a typical experiment, $PbBr_2$ (3.67 g, 10 mmol), oleic acid (18.75 mL), oleylamine (18.75 mL), and octadecene (50 mL) were added to a two-neck round-bottomed flask (100 mL). The mixture was heated to 120 °C in vacuo for 0.5 h with vigorous stirring and stirred at 120 °C for another 0.5 h in the same environment. After that, the moisture residue of the flask was removed by alternately purging with nitrogen and vacuum suction three times. The mixture was then heated to 160 °C and became clear, indicating that the $PbBr_2$ precursors had dissolved completely. Hot Cs-oleate precursor solution (5 mL, 0.5 mmol) was swiftly injected into the above reaction mixture when it was heated to 180 °C. After 10 s of reaction, the flask was transferred to an ice bath and cooled to 50 °C in a nitrogen environment. The $CsPbBr_3$ nanocrystals were obtained by centrifugation at 9000 r.p.m. for 2 min and stored in toluene (6 mL) before further use.

Synthesis of AHFS-$CsPbBr_3$ nanocrystals. An aqueous solution of AHFS (200 µL, 0.5 M) was added to a toluene solution of $CsPbBr_3$ nanocrystals (15 mL, 5 mg/mL). To ensure that AHFS and $CsPbBr_3$-nanocrystals

were in full contact, the mixture was then stirred for 1 h. Subsequently, the denser water phase was separated by high-speed centrifugation at 9000 r.p.m. for 2 min. The sedimentation was Then dissolved in toluene (15 mL), and AHFS water solution (200 μL, 0.5 M) was added with stirring (2 h). Afterward, the water phase was separated by high-speed centrifugation at 9000 r.p.m. for 2 min. After removing the upper-layer solution containing unreacted AHFS, the AHFS-$CsPbBr_3$ nanocrystals were obtained from the sedimentation for further characterization.

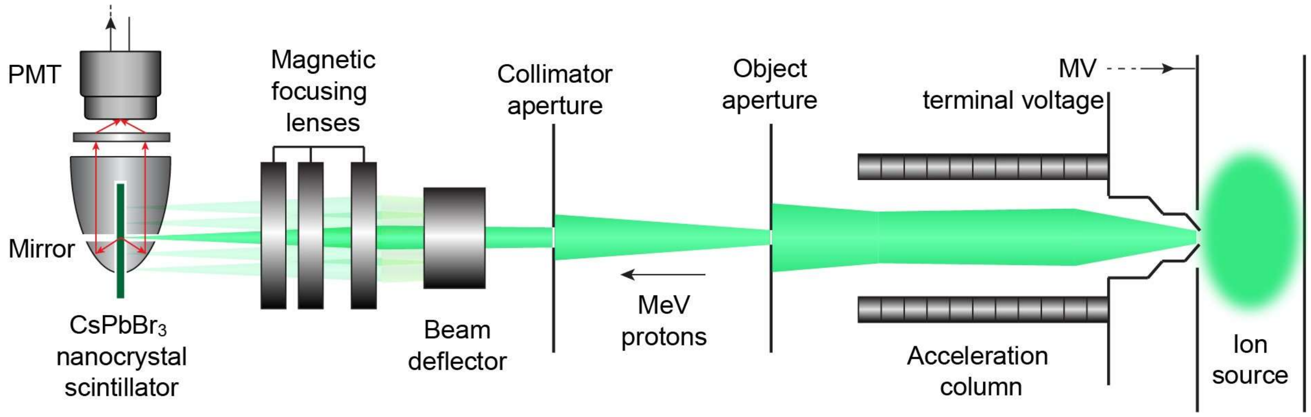

Supplementary Fig. 1 | Instrumentation for the generation of a focused proton beam and the collection of luminescence. Schematic showing the generation of MeV protons using an ion accelerator equipped with a hydrogen ion source, the basic beam optics system comprising an objective aperture, a collimating aperture and a spaced triplet of magnetic quadrupole lenses for the formation of a focused proton beam, and the experimental setup for light collection and detection using a custom-built aluminum parabolic mirror and a photomultiplier tube (PMT).

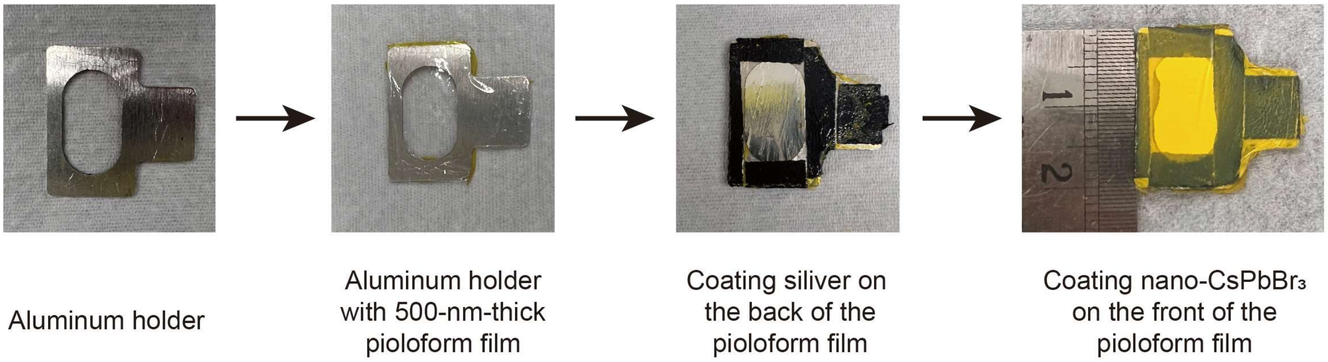

Supplementary Fig. 2 | Preparation of thin-film perovskite nanocrystal scintillators. From left to right, images of a custom-hollowed-out aluminum holder (thickness, 500 μm), a homemade pioloform film (thickness, 500 nm) prepared on the aluminum holder, a silver coating on the back side of the pioloform film to enhance the reflectivity of the scintillation light, and a scintillator film comprising $CsPbBr_3$ nanocrystals coated on the front side of the pioloform film. The measurement shows a length of about 20 mm of the as-prepared scintillator film.

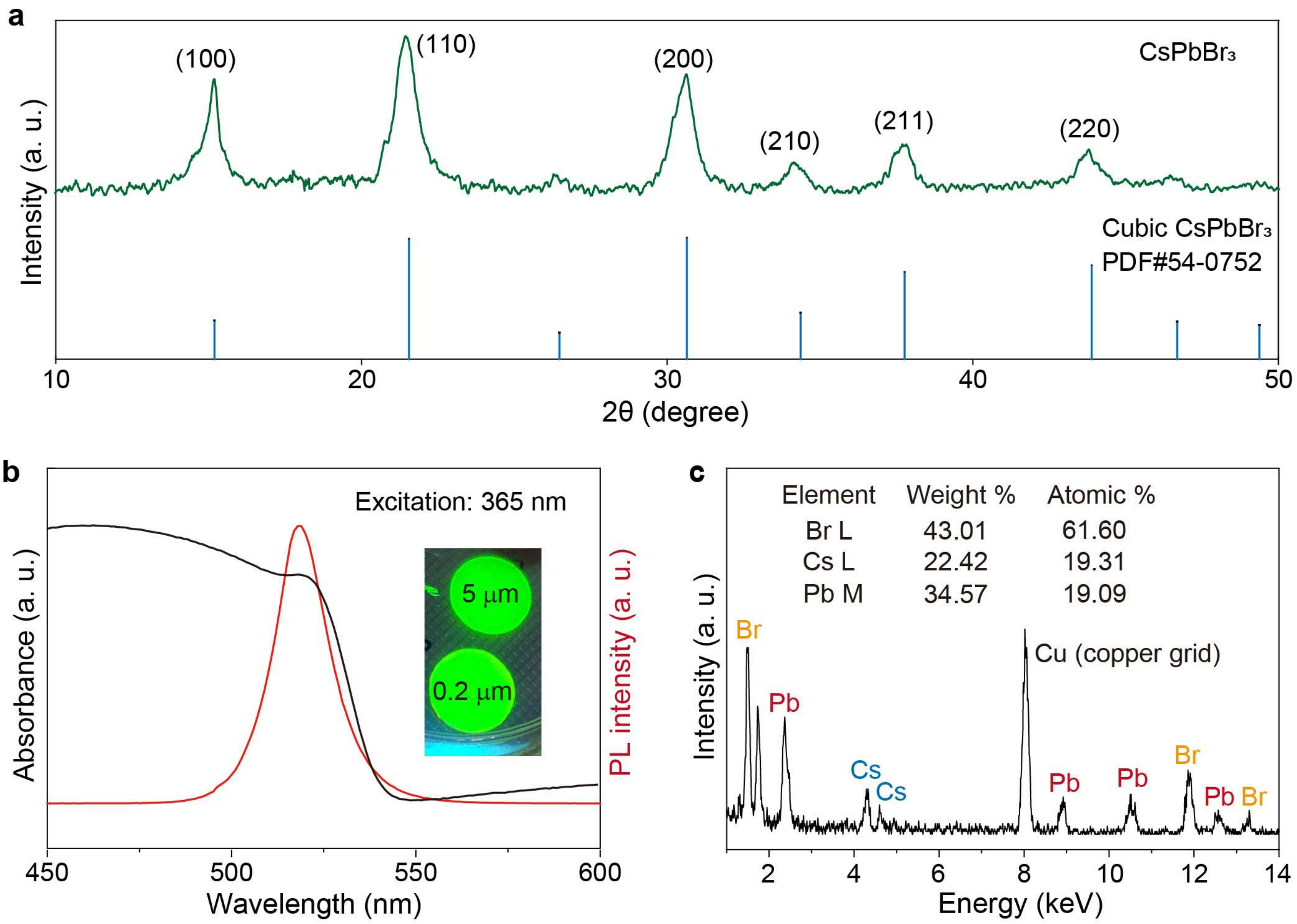

Supplementary Fig. 3 | Physical characterization of as-synthesized perovskite nanocrystals. a, Powder X-ray diffraction pattern of $CsPbBr_3$ nanocrystals. All peaks are indexed in accordance with the cubic-phase $CsPbBr_3$ structure (Joint Committee on Powder Diffraction Standards file (PDF) number 54-0752). **b**, Absorbance (left axis) and photoluminescence (PL, right axis) spectra of $CsPbBr_3$ nanocrystals (excitation wavelength, $\lambda_{ex} = 365$ nm). The inset image shows the scintillation of two $CsPbBr_3$ nanocrystal scintillators (thicknesses, 0.2 μm and 5 μm, respectively) prepared on quartz glass, upon illumination with a 365-nm light source. **c**, Energy dispersive X-ray spectrum of the as-prepared $CsPbBr_3$ nanocrystals, confirming the stoichiometric composition of the $CsPbBr_3$ nanocrystals. Note that the strong Cu signal results from the TEM holder of the copper grid.

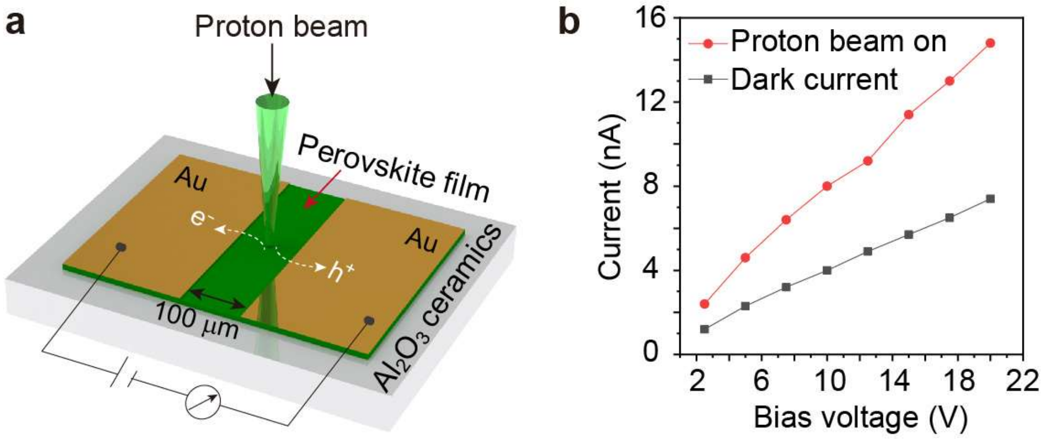

Supplementary Fig. 4 | Experimental verification of charge carrier production in the perovskite scintillator upon proton irradiation. a, Schematic of the basic experimental design for the detection of electron-hole carriers produced by a proton beam within a perovskite scintillator. A thin layer of $CsPbBr_3$ nanocrystals (50-µm-thick) was coated onto the Al_2O_3 ceramic substrate to receive the energy deposition by protons. Two gold electrodes (50-nm-thick) were sputtered onto the scintillator film to collect charge carriers. **b**, Current-voltage measurements of the as-fabricated charge carrier detector with and without illumination by 2 MeV proton beam.

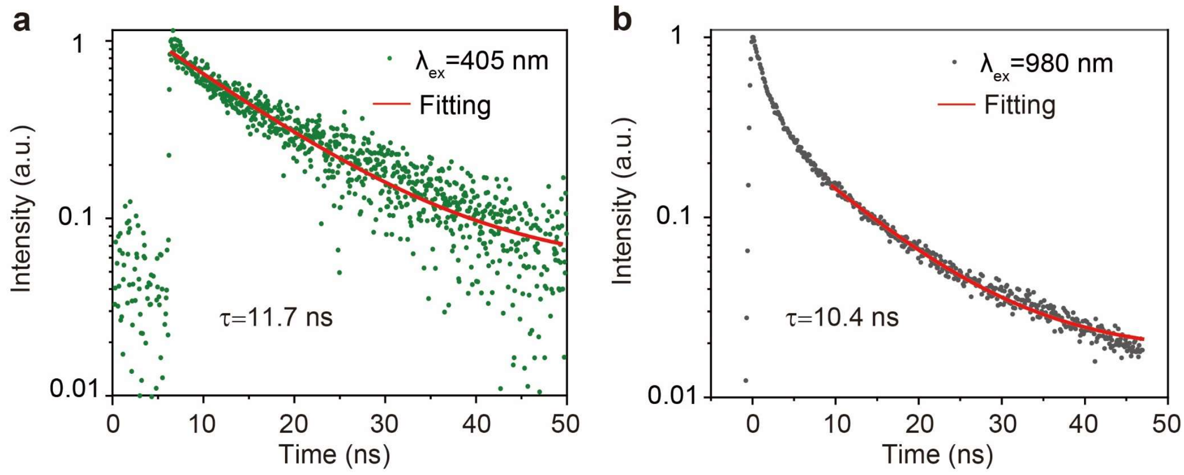

Supplementary Fig. 5 | Photoluminescence lifetime measurement of $CsPbBr_3$ nanocrystals. a, Photoluminescence decay profile under 405-nm excitation, showing a lifetime of 11.7 ns. **b**, Photoluminescence decay profile under 980-nm excitation, showing a lifetime of 10.4 ns. Note that single-exponential decay functions were used for curve fitting in **a** and **b**.

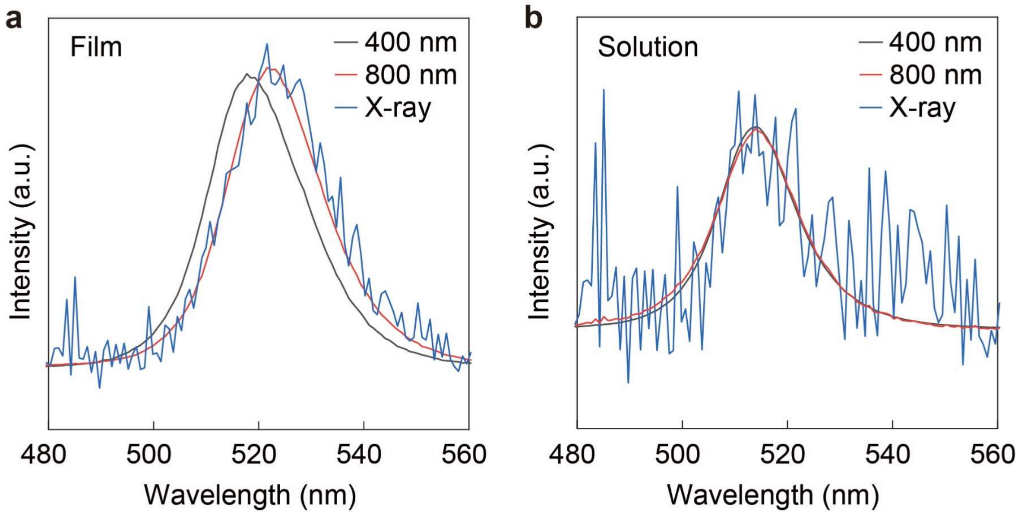

Supplementary Fig. 6 | Emission spectra of $CsPbBr_3$ nanocrystals under different excitation sources. a, $CsPbBr_3$ nanocrystals prepared in the form of a film, showing a red-shifted emission under excitation by 800-nm and X-ray photons compared with emission under 400-nm excitation. **b**, $CsPbBr_3$ nanocrystals dissolved in toluene, showing no red shift under 400-nm, 800-nm, or X-ray excitation.

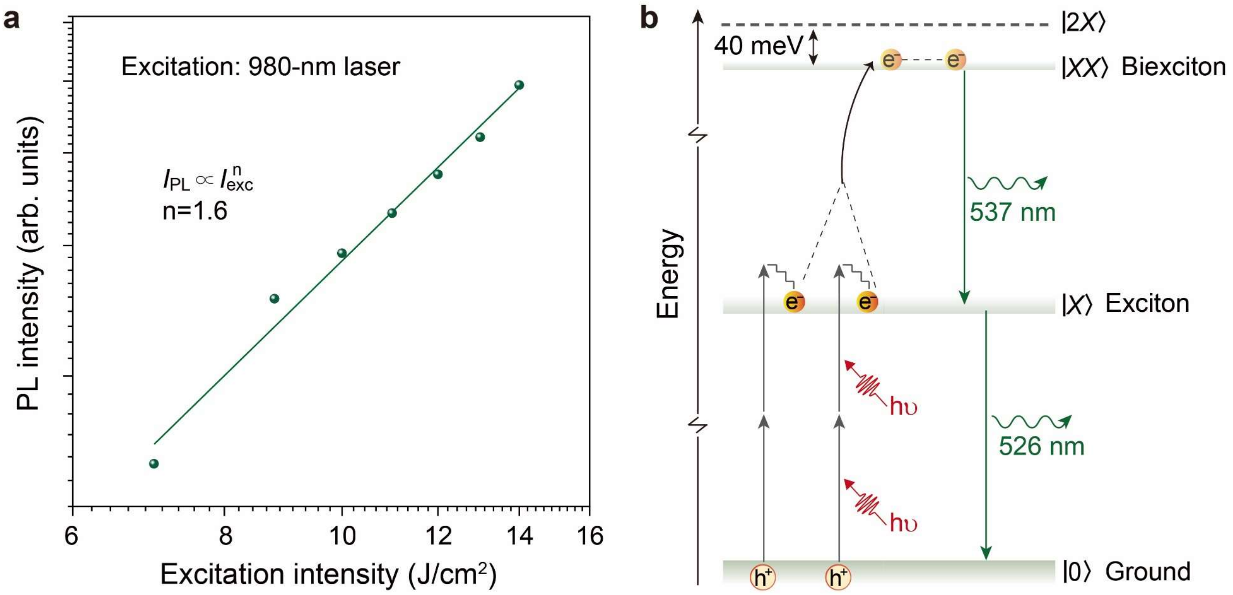

Supplementary Fig. 7 | Upconversion photoluminescence through below-bandgap multiphoton absorption in perovskite nanocrystals. a, Photoluminescence (PL) intensity (I_{PL}) as a function of excitation intensity (I_{exc}), indicating a 2-photon absorption process in $CsPbBr_3$ nanocrystals under excitation with a 980-nm laser. **b**, Proposed mechanism of upconversion photoluminescence under 980-nm excitation. Multiple exciton generation occurs through absorption of two 980-nm photons and rapid thermalization. Two excitons are subsequently converted to form a biexciton. De-excitation from the biexcitonic state to the excitonic state gives rise to emission at 537 nm, which dominates the PL emission. As a result, the emission is red-shifted (~40 meV) in contrast to the emission due to de-excitation from the excitonic state (~526 nm).

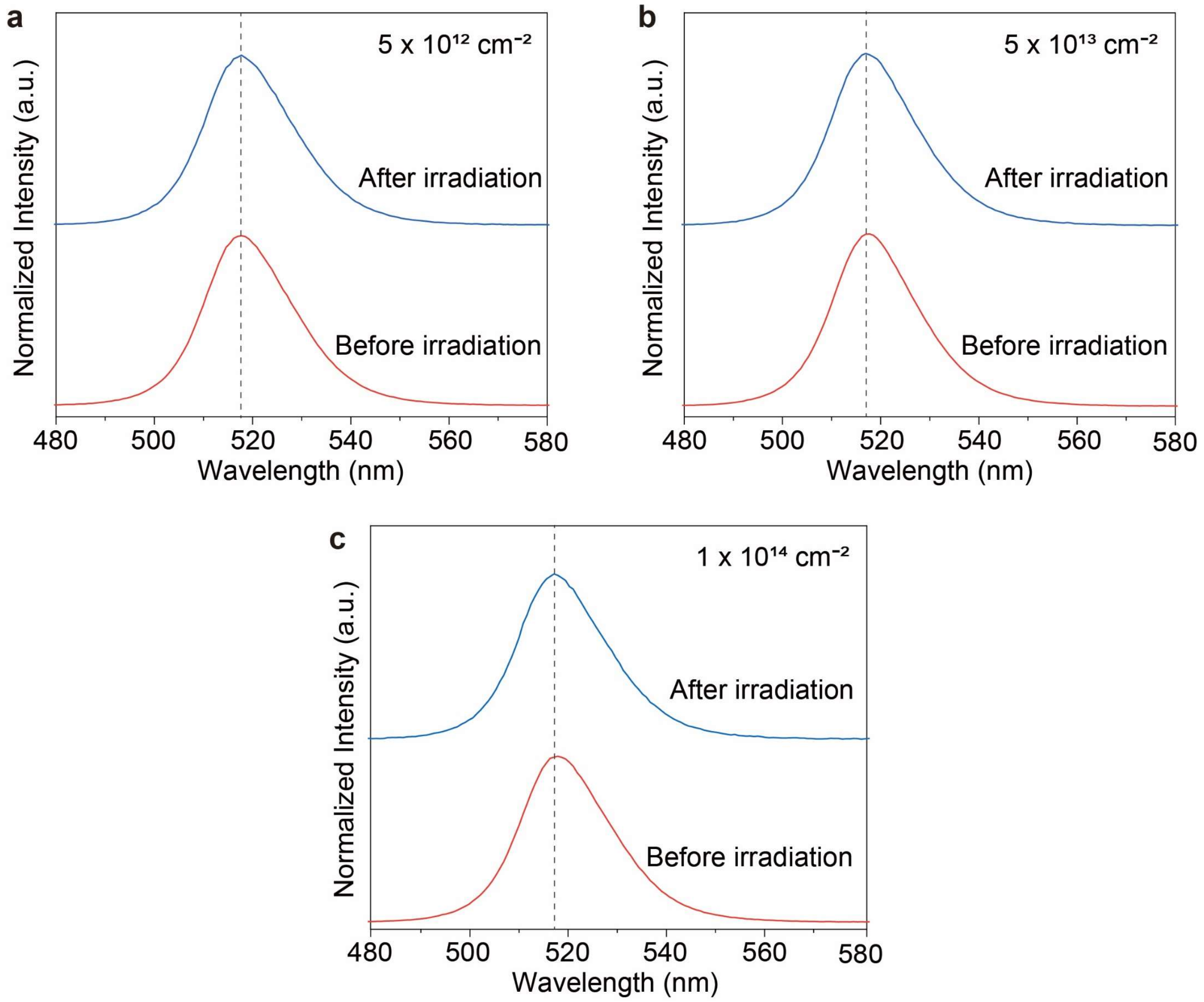

Supplementary Fig. 8 | Effect of proton irradiation on the photoluminescence emission from $CsPbBr_3$ nanocrystals. In the context of the radiation tolerance of $CsPbBr_3$ nanocrystals to protons (fluence~1×10^{14} cm^{-2}), we irradiated three areas of a $CsPbBr_3$ thin film with a proton fluence of **a**, 5×10^{12} cm^{-2}, **b**, 5×10^{13} cm^{-2}, and **c**, 1×10^{14} cm^{-2}, respectively. For each area, we performed photoluminescence (PL) measurements with 400-nm excitation before and after proton irradiation. The PL emission spectra remained unaltered and red shifts due to proton irradiation were not observed. The energy of the protons used was 2 MeV.

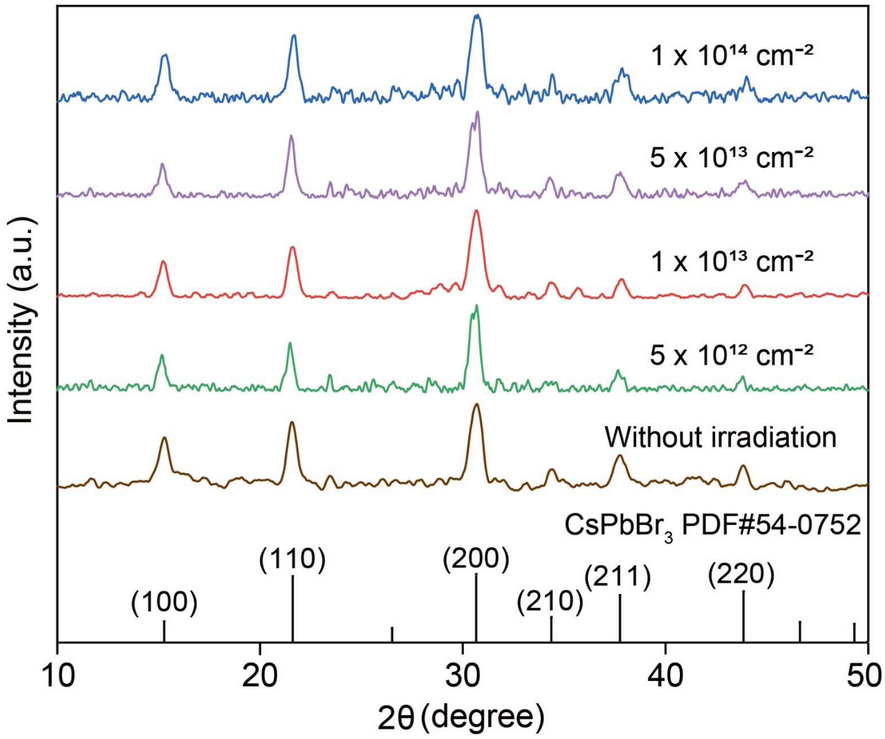

Supplementary Fig. 9 | X-ray diffraction characterization of $CsPbBr_3$ nanocrystals with and without proton irradiation. A $CsPbBr_3$ thin film was irradiated in different areas with 2 MeV protons with fluence of 5×10^{12} cm^{-2}, 1×10^{13} cm^{-2}, 5×10^{13} cm^{-2}, and 1×10^{14} cm^{-2}, respectively. The irradiated areas were then characterized in situ with X-ray diffraction (XRD). For comparison, an area of the $CsPbBr_3$ thin film without proton irradiation was selected to perform XRD measurements as well. All diffraction peaks can be well indexed in accordance with the $CsPbBr_3$ crystal structure (Joint Committee on Powder Diffraction Standards file No. 54-0752). The results indicate that no defect-related structure was formed under proton irradiation.

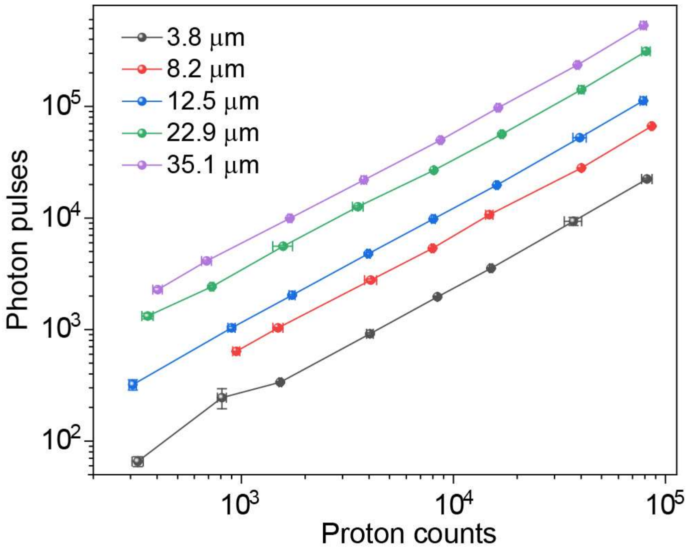

Supplementary Fig. 10 | Single-proton counting with $CsPbBr_3$ nanocrystal thin films. The detected ionoluminescence photon-pulse counts were recorded in terms of the corresponding proton counts for $CsPbBr_3$ nanocrystal thin films with different thicknesses (3.8, 8.2, 12.5, 22.9, and 35.1 μm). The results indicate that the photon-pulse counts are linearly proportional to the proton counts within a measurement range of 10^2~10^5 protons. The energy of the proton beam used was 2 MeV. n = 3 independent experiments. Data are presented as mean values ± standard error of the mean (s.e.m.).

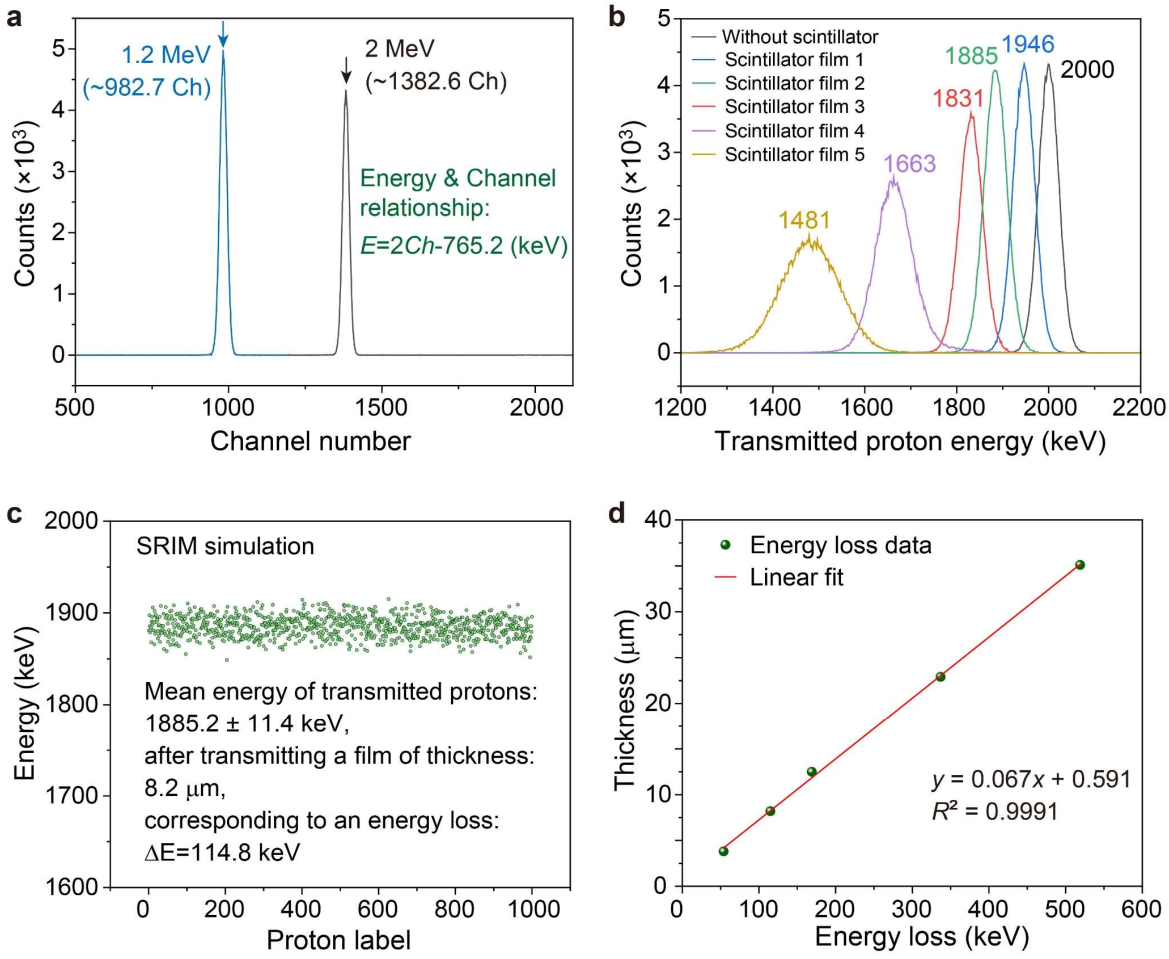

Supplementary Fig. 11 | Thickness measurement of perovskite scintillator films. a, Calibration of ADC channel number to energy. Energy-resolved spectra of two proton beams with different energies (1.2 and 2 MeV, respectively) were first collected via a silicon detector. The relationship between the energy (*E*) and the channel number (*Ch*) was then determined by calibrating these two specific proton energies and their corresponding channel numbers. **b**, Energy-resolved spectra of transmitted protons after penetrating scintillator films of 5 different thicknesses. Note that the spectrum with a 2000-keV peak was obtained directly via the silicon detector without the protons having penetrated any scintillator films. **c**, A representative simulation using the SRIM software to determine the thickness of the scintillator film (~8.2 μm) through the energy loss (ΔE) of protons in the film. Note that 1000 protons were simulated. **d**, Thickness of scintillator films as a function of the corresponding energy loss of the protons in the films. The fit indicates a linear relationship between the thickness and the energy loss.

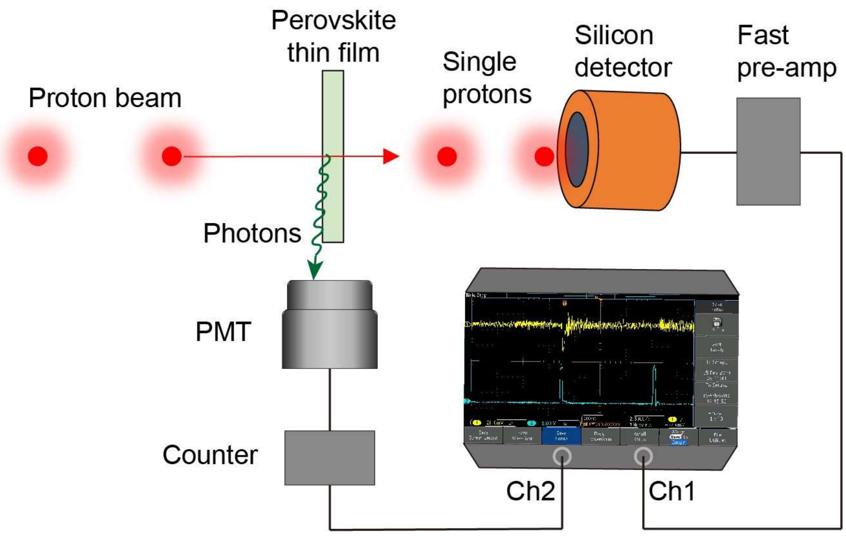

Supplementary Fig. 12 | Single-proton tracing in $CsPbBr_3$ nanocrystal thin films. Schematic showing the basic experimental setup for single proton tracking and the resulting photon pulses. The count rate of incident protons is kept low (~500 s^{-1}) to ensure that only one single proton is detected within a certain time domain. A single proton transmits the perovskite thin film and is detected by a silicon surface barrier detector (Ortec). The single-proton signal is subsequently amplified to about 30 mV with a fast pre-amplifier (cividec Instrumentation, Diamond TCT Amplifier, 2 GHz, 40 dB) and captured by a fast oscilloscope (Tektronix MDO3024) through Channel 1 (Ch1). The corresponding ionoluminescence photons induced by this single proton are detected with a PMT (Hamamatsu R7400P) equipped with a photon counting unit (Hamamatsu C9744), and the signal of photon pulses is captured by the oscilloscope through Channel 2 (Ch2).

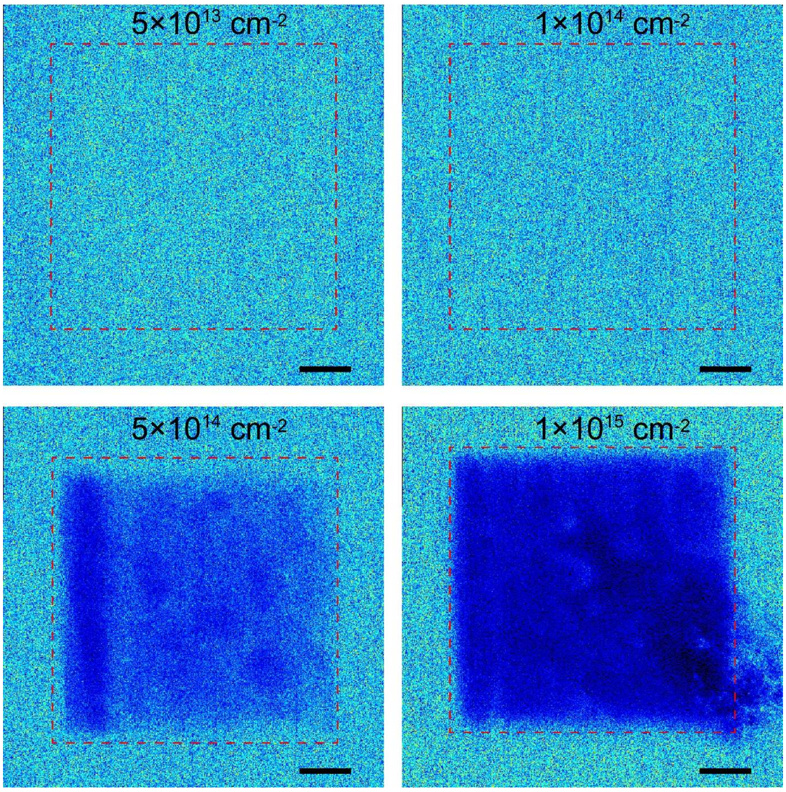

Supplementary Fig. 13 | Radiation tolerance of a 50-μm-thick perovskite scintillator to protons. Four regions of the scintillator with the same area (110 μm × 110 μm) were implanted with a proton fluence of 5×10^{13}, 1×10^{14}, 5×10^{14}, and 1×10^{15} cm^{-2}, respectively. After proton implantation, each region was imaged by collecting ionoluminescence photon counts pixel by pixel. The proton-implanted regions are marked in the four raw ionoluminescence images. Note that the proton fluence for ionoluminescence imaging was 5×10^{10} cm^{-2} which is negligible compared with the fluence for implantation. These results indicate that the ionoluminescence intensity decreases significantly only at a proton fluence of 5×10^{14} cm^{-2}. Proton energy, 2 MeV. Scale bars are 20 μm.

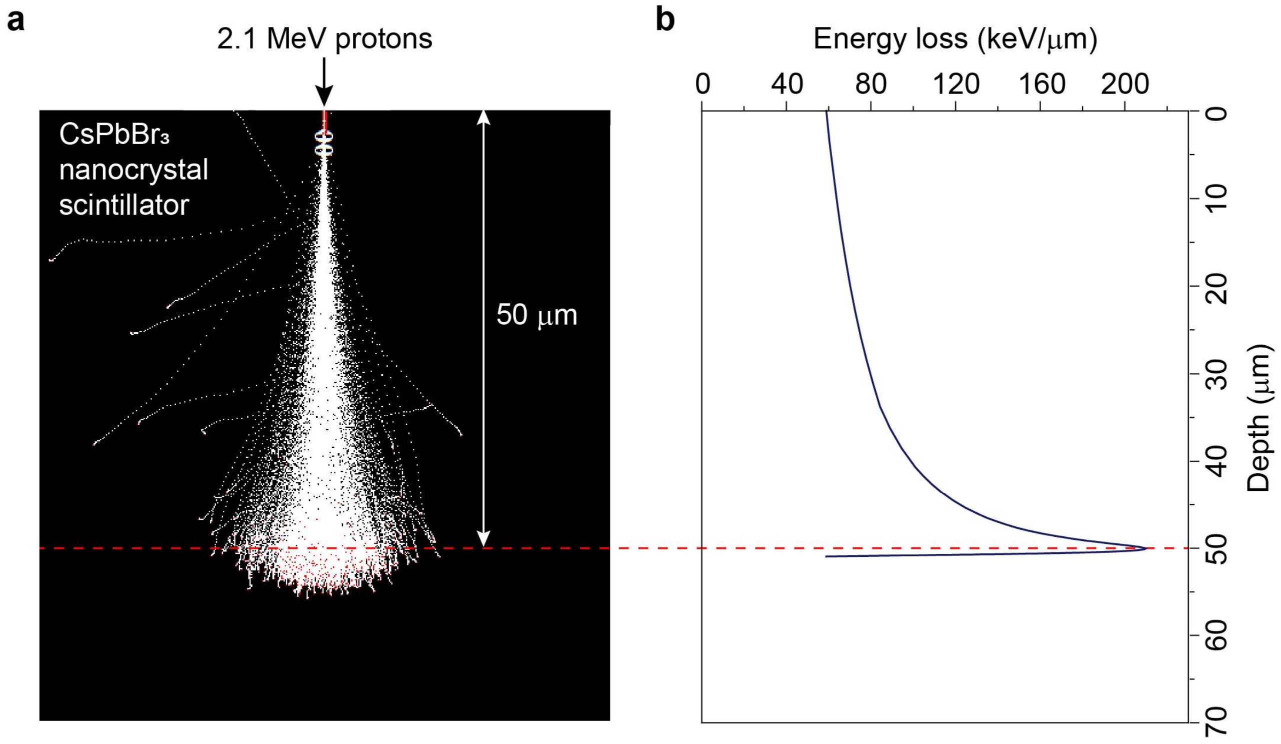

Supplementary Fig. 14 | Monte Carlo simulation of the range and stopping of 2.1 MeV protons in $CsPbBr_3$ nanocrystal films using SRIM. a, Image showing simulated trajectories of protons in the scintillator. In this case, 1000 protons were used for the simulation. The result indicates a penetration depth of 50 µm of 2.1-MeV protons into the $CsPbBr_3$ nanocrystal scintillator. **b**, Stopping power per proton as a function of proton depth. The maximum energy transfer occurs at the end-of-range of protons. In this case, 10000 protons were used for the simulation.

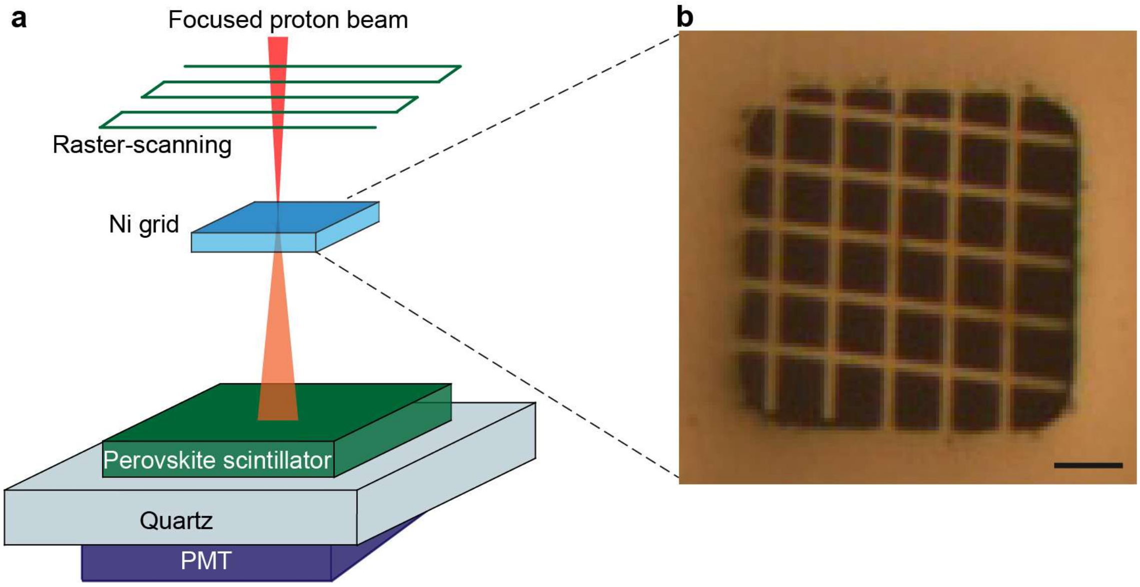

Supplementary Fig. 15 | Proton imaging with $CsPbBr_3$ scintillators. a, Schematic of the basic experimental setup for proton imaging with a $CsPbBr_3$ scintillator. A focused proton beam scans across a home-made Ni calibration grid with a raster-scanning pattern. The transmitted protons are detected by the bulk perovskite scintillator (thickness: ~120 μm) coated on a quartz glass. The scintillating ionoluminescence photons are detected by a photomultiplier tube (PMT). **b**, Optical image of the Ni calibration grid used for proton imaging. Scale bar, 20 μm.

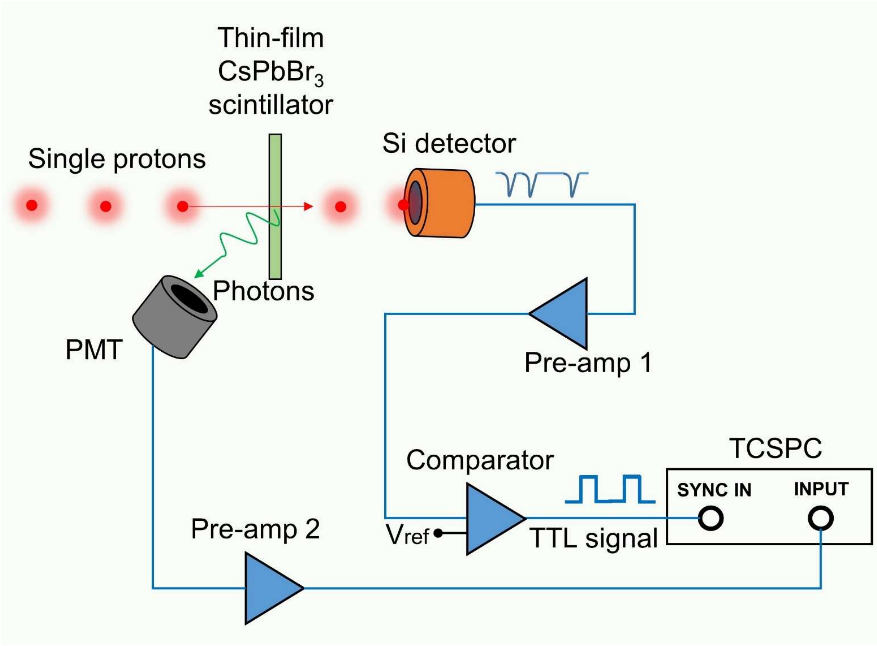

Supplementary Fig. 16 | Time-resolved ionoluminescence measurements. Schematic of the basic experimental setup for ionoluminescence lifetime measurement. A single proton that transmits the thin-film $CsPbBr_3$ scintillator is detected by a silicon surface barrier detector (Ortec). The signal from this single proton is amplified by a fast pre-amplifier (cividec Instrumentation, Diamond TCT Amplifier, 2 GHz, 40 dB), labeled 'Pre-amp 1'. The amplified single-proton signal is then captured by a fast oscilloscope (Tektronix MDO3024), which is used as a comparator to synchronize the time-correlated single-photon counting (TCSPC) hardware (PicoQuant Timeharp 260 PICO) by generating a transistor-transistor logic (TTL, 5 V) signal. The corresponding ionoluminescence photons induced by this single proton are detected by a hybrid photomultiplier detector (PicoQuant PMA Hybrid 40). The photon signal is then amplified by the second pre-amplifier, marked as 'Pre-amp 2'. The amplified photon signal is acquired and processed by the TCSPC card to register the photon arrival time.
